# G-protein signaling components differentially regulate the activity of multiple cellulase enzyme classes in *Neurospora crassa*

**DOI:** 10.1186/s13068-026-02779-x

**Published:** 2026-06-05

**Authors:** Yagna Oza, Katherine A. Borkovich

**Affiliations:** 1 Department of Microbiology and Plant Pathology, University of California, Riverside, CA 92521 USA; 2 Present Address: Department of Biobehavioral Health, Pennsylvania State University, University Park, PA 16802 USA

**Keywords:** Cellulases, Environmental sensing, Genetic engineering, *Neurospora crassa*, Filamentous fungi

## Abstract

**Background:**

Heterotrimeric G-protein subunits, adenylyl cyclase, and Regulators of G-protein Signaling (RGS) proteins are required for the conversion of cellulose to glucose in the model filamentous fungus *Neurospora crassa*. Transcriptomics has revealed roles for several of these genes in the expression of specific cellulase enzyme classes, including endo-β-1,4-glucanases (endoglucanase), exo-β-1,4-glucanases (cellobiohydrolases), β-1,4-glucosidases (β-glucosidases), polysaccharide monooxygenases and cellobiose dehydrogenases.

**Results:**

In this study, we measured endoglucanase, cellobiohydrolase and β-glucosidase enzyme activities in secretomes from all G-protein subunit and RGS single mutants, Gα multiple mutants, and the adenylyl cyclase mutant Δ*cr-1* after transfer from glucose to cellulose medium. We observed that the Gα subunits *gna-1* and *gna-3* together are required for endoglucanase activity. *cr-1* is essential, while *gna-2* synergizes with *gna-3* to regulate cellobiohydrolase activity. Multiple G-protein genes impacted β-glucosidase activity to some extent, but none were essential for activity. cAMP supplementation of the G protein mutants resulted in the greatest increase in endoglucanase and cellobiohydrolase activity, suggesting an important role for cAMP levels in the activity of these enzyme classes. Among the RGS mutants, the Δ*rgs-2* strain has greatly reduced activity for all three enzyme classes, like the strain expressing the constitutively active *gna-3*^Q208L^ allele. In contrast, the Δ*rgs-1* mutant and the *gna-1*^Q204L^ strain showed significantly higher endoglucanase activity as compared to wild type.

**Conclusions:**

Taken together, our results indicate that G protein signaling components differentially impact the activity of three major cellulase enzyme classes in *N. crassa*. Our findings will inform future strain engineering approaches to increase production of specific cellulase activities in culture supernatants of filamentous fungi.

**Supplementary Information:**

The online version contains supplementary material available at 10.1186/s13068-026-02779-x.

## Background

Plant cell walls are made up of lignocellulolytic materials, including the most abundant carbon polymer on earth, cellulose. Cellulose is composed of glucose molecules joined by β−1,4-linkages, with weak hydrogen bonds linking chains in the structure [27, 30]. Pathogenic and saprophytic fungi secrete plant cell wall degrading enzymes (PCWDEs) and utilize the degradation products of cellulose such as cellobiose (glucose dimer) and glucose as carbon sources [15]. Efficient degradation of cellulose by the lignocellulose degrading enzymes produced by microbial organisms is a major economic bottleneck in the biofuel industry [46]. The fungus *Trichoderma reesei* has been extensively modified for increased industrial cellulase production [4, 32], and numerous cellulose degradation regulatory genes have been identified in other fungi such as *Neurospora crassa* [12, 16, 25, 43, 44]. However, identification of upstream environmental sensing pathways that modulate cellulase production is an understudied area.

Filamentous fungi use heterotrimeric G-protein signaling to sense carbon sources in the environment [10, 24, 33, 34]. The G-protein heterotrimeric complex is composed of α, β, and γ subunits. Ligand sensing by a G protein coupled receptor (GPCR) leads to the exchange of bound GDP for GTP on the Gα subunit of the heterotrimer and dissociation of the Gα-GTP and Gβγ dimer, leading to the regulation of downstream effectors [26]. Hydrolysis of GTP to GDP by the Gα subunit returns the signaling cascade to its inactive state. The filamentous fungus *Neurospora crassa* possesses three Gα subunits (GNA-1, GNA-2, and GNA-3), two Gβ subunits (GNB-1 and CPC-2), and one Gγ subunit (GNG-1) [14, 20, 23, 38, 45]. One of the major pathways identified downstream of G-proteins in *N. crassa* is cAMP signaling. Adenylyl cyclase (CR-1) activity, protein quantity and cAMP levels are influenced by G-proteins in *N. crassa* [17, 19, 20, 45]. Recent work from our research group showed that the GNA-1 and GNA-3, GNB-1, GNG-1 and adenylyl cyclase also play a crucial role in degradation of crystalline cellulose (Avicel) to glucose (Avicelase activity) in *N. crassa* [10].

Regulator of G-protein Signaling (RGS) proteins negatively control the activity of Gα subunits by accelerating the rate of GTP hydrolysis by up to 2000 times [29]. RGS proteins have been characterized in multiple eukaryotic systems, including fungi [1, 5, 18, 47]. Our group characterized functions for all seven *N. crassa* RGS genes during hyphal growth, asexual and sexual development, and cellulose degradation [6]. Perhaps the most striking observation was an increase in Avicelase activity for the ∆*rgs-1* mutant and a strain expressing a GTPase-deficient allele of *gna-1* (*gna-1*^Q204L^), while there was no activity detected for the ∆*rgs*−2 mutant and the corresponding *gna-3*^Q208L^ strain, carrying a GTPase-deficient allele of *gna-3*. This suggested an interaction between GNA-1 and RGS-1 and GNA-3 and RGS-2 in *N. crassa* during growth on cellulose [6].

**Fig. 1:**
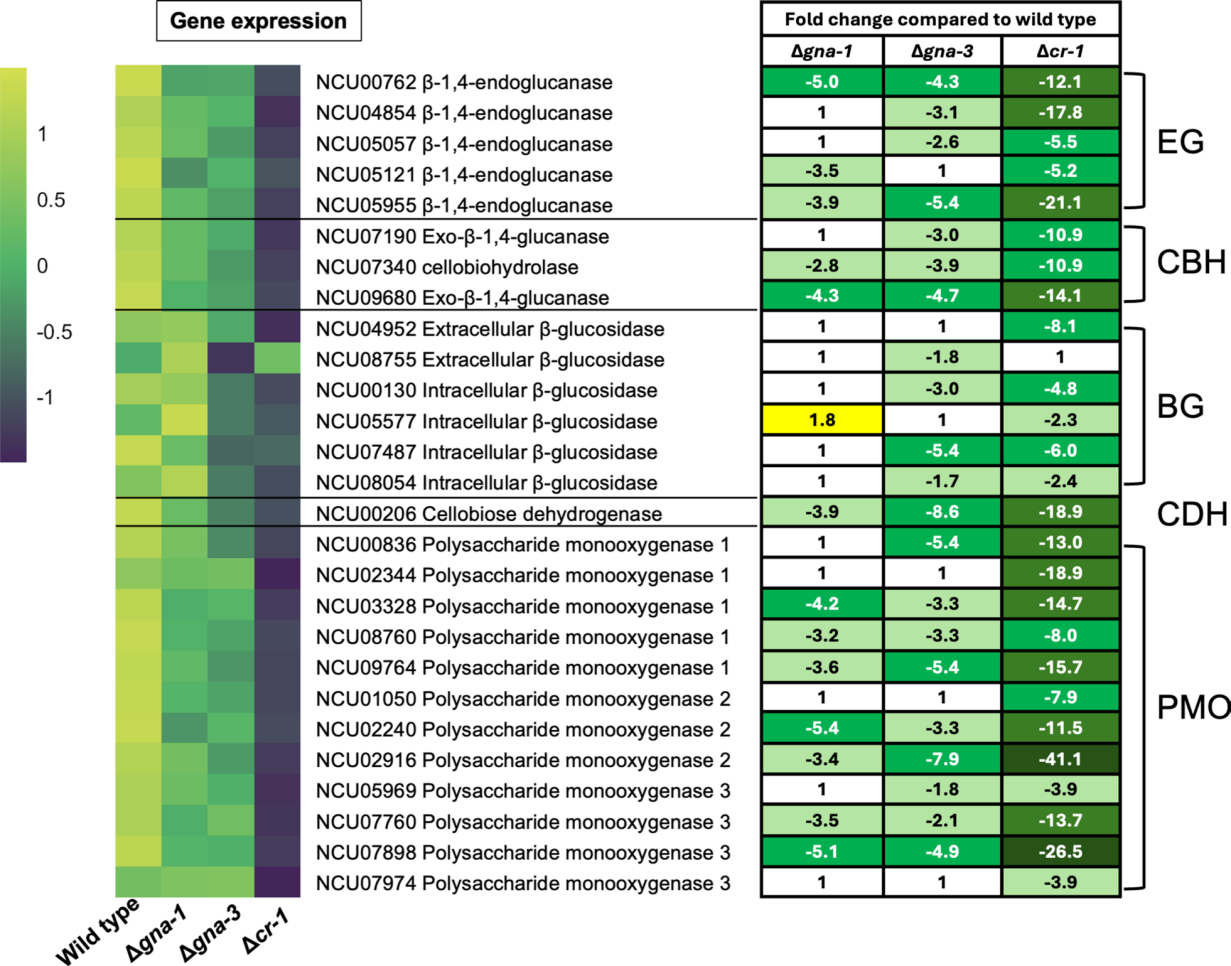
Hierarchical clustering of Transcripts Per Million and DESeq2 data for 27 cellulase genes in the wild type, Δ*gna-1*, Δ*gna-3* and Δ*cr-1* strains during growth on cellulose. The average Transcripts Per Million (TPMs) for cellulase genes obtained from RNAseq data from a previous study [9] were Log2 transformed and used to construct a heatmap using hierarchical clustering (left). All strains were cultured on glucose for 12 h, washed in no-carbon medium, and then transferred to medium containing 2% Avicel for 4 h prior to RNA extraction [9]. Yellow and dark green shading indicate higher and lower expression levels for a given gene, respectively. The maximum relative level was 1.5 (brightest yellow) and the minimum relative level was −1.5 (darkest green). The table on the right presents DESeq2 fold changes relative to wild type during growth on cellulose obtained from the same study [9]. The same shading is used as for the heatmap, except that values are relative to wild type

The *N. crassa* genome encodes cellulase enzyme genes from several families, including endoglucanases, cellobiohydrolases, β-glucosidases, cellobiose dehydrogenases, and polysaccharide monooxygenases [15]. Endoglucanases cleave the cellulose chain internally using a hydrolysis reaction [7]. In contrast, polysaccharide monooxygenases create open ends in cellulose through oxidative cleavage in the presence of an electron donor (cellobiose) through cellobiose dehydrogenase [3, 28, 35]. The cellobiohydrolase enzymes release cellobiose molecules from the open ends, and the β-glucosidase enzymes hydrolyze the β−1,4-linkages in cellobiose to release two glucose molecules [2, 31, 39] (Fig. 1). Most cellulolytic enzymes are secreted by *N. crassa* outside the cell, there are, however, several intracellular β-glucosidases that hydrolyze cellobiose that has been transported inside of the cell [15].

In *N. crassa*, cellulose degradation is regulated by five regulatory proteins termed cellulose degradation regulators (CLRs) [11, 13, 16, 25, 44]. The zinc binuclear cluster transcription factors CLR-1 and CLR-2 are essential for the breakdown of cellulose into β−1,4-linked glucose oligosaccharides and eventually into glucose [11]. The presence of intracellular cellobiose induces c*lr-1* expression, and cellobiose is also thought to activate the CLR-1 protein [48]. Activated CLR-1 induces the transcription of *clr-2*, and CLR-2 activates expression of numerous cellulase genes [11, 41]. CLR-1 also regulates the expression of several carbon transporter and β-glucosidase genes [11].

We recently performed RNAseq on wild type, Δ*gna-1*, Δ*gna-3* and Δ*cr-1* mutants after transfer from glucose to cellulose [9]. We observed that expression of *clr-2,* as well as all cellulase families except for β-glucosidases, are reduced in Δ*gna-1* mutants. In contrast, Δ*gna-3* mutants had lower expression of *clr-1* and *clr-2* and exhibited more severe downregulation of all cellulase enzyme families. The Δ*cr-1* mutant had the greatest defect, with reduced levels of *clr-1* and *clr-2*, and cellulase genes were greatly downregulated. Transformation of wild type and the three mutants with a construct to overexpress *clr-2* [12] led to increased Avicelase activity, reinforcing the notion that *clr-2* operates downstream of *gna-1*, *gna-3* and *cr-1* [9].

One of our goals is to identify the environmental sensing genes that regulate cellulase production and then to exploit this information to engineer genetic backgrounds with increased cellulase activity. Although Avicelase activity provides information about overall misregulation of cellulases, it is not possible to estimate how each cellulase family contributes to the resultant activity. We were interested in determining the impact that transcriptional control of cellulase enzyme genes may have on the levels of the respective enzymes in various G-protein signaling mutants [9]. To further this goal, we examined the G-protein, adenylyl cyclase, and RGS gene deletion mutants for their endoglucanase, cellobiohydrolase, and β-glucosidase activities after transfer from glucose to cellulose. We elucidated possible coregulation of specific cellulase families by multiple Gα subunits using strains lacking 2–3 Gα genes. Our results demonstrate several examples of cooperative regulation of the three enzyme classes by G protein signaling genes and illuminate future strategies to increase secretion of these enzymes.

## Methods

### Strains and growth conditions

Strains are listed in Table S1. Conidia were harvested from agar flasks containing Vogel’s minimal medium [40] and used to inoculate cultures. Liquid VM cultures (25 ml) with 2% glucose as the carbon source were inoculated using 1 × 10^6^ conidia/ml and grown for 16 h in constant light at 25 ºC with shaking at 200 RPM [10]. Cultures were washed twice with VM medium without a carbon source and then transferred to VM liquid medium with 2% Avicel (cellulose) to grow for 48 h [9]. Where indicated, glucose and Avicel media were supplemented with cAMP at 3 mM (#A6885, Sigma-Aldrich, St. Louis, MO USA). Cell-free supernatants (secretome samples) were collected using a syringe and passed through a 0.45 micron filter to remove cells, as previously described [10]. Secretome samples were concentrated and subjected to SDS-PAGE analysis as described [10]. Cell pads were collected using vacuum filtration and washed in water to remove any residual secretions. Total cellular protein (biomass protein) was extracted from the cell pads using a buffer containing SDS, with heating and agitation using glass beads as previously described [10].

### Hierarchical clustering analysis of RNAseq data

Transcripts per Million (TPMs) for a group of 27 annotated and expressed cellulases in wild type, Δ*gna-1*, Δ*gna-3* and Δ*cr-1* mutants during growth on cellulose were obtained from a previous study [9]. Hierarchical clustering analysis was conducted using Euclidean distance as the similarity metric and the complete linkage method for cluster generation using pheatmap (V1.0.13) [22] in R [36].

### Protein and enzyme assays

The protein concentration in the cell pad extracts and the cell-free supernatants was measured using the Pierce Bicinchoninic acid assay (Thermo Fisher Scientific, Chino, CA, USA), with bovine serum albumin as the standard. The ability of the cell-free secretomes to degrade Avicel all the way to glucose was determined using the Avicelase assay as previously described [10]. Endoglucanase activity was measured using the substrate Azo-CMC-cellulase (S-ACMC, #700005041) as recommended by the manufacturer (Megazyme/Neogen, Lansing, MI, USA), except using one-fifth volumes for all assay reagents. The cellobiohydrolase assay [44] was performed by using up to 90 µl of cell-free supernatant in a 500 µl reaction mix consisting of the substrate *p-*nitrophenol-β-D-cellobioside (#N5759-100MG, Sigma-Aldrich) in 0.1 M sodium citrate buffer, pH 5.0. Reactions were incubated at 37 ºC for 20 min. The reaction was quenched using 625 µl of 0.1 M sodium hydroxide and absorbance was measured at 405 nm. The same protocol was followed for the β-glucosidase assay [44] except that the substrate was *p-*nitrophenol-β-D-glucopyranoside (#N81090-1.0, Research Products International, Mount Prospect, IL, USA) and the incubation time at 37ºC was 15 min.

### Statistical analysis

**Fig. 2:**
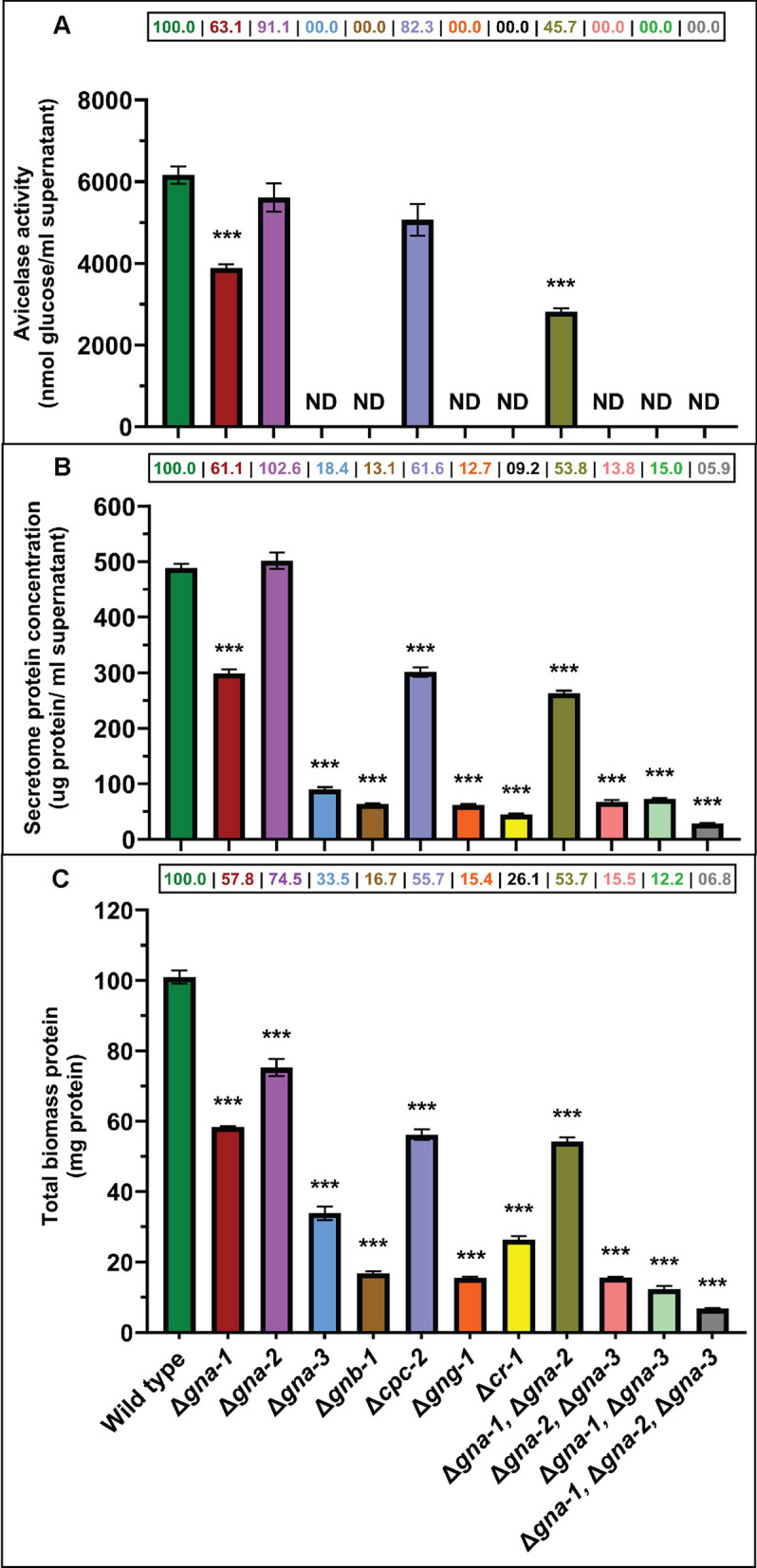
Avicelase activity and secretome and biomass protein levels in G-protein and adenylyl cyclase mutants. Strains were cultured in VM glucose medium for 16 h. Mycelia were collected and washed twice using VM no carbon medium, and then transferred to VM Avicel medium for 48 h. A sample of each culture supernatant was withdrawn and passed through a 0.45-micron filter. A minimum of three replicates were used for all assays, and errors are expressed as the standard error. Statistical significance relative to the wild type was determined using a two-tailed Welch’s t-test, and strains with p-values indicated as *p < 0.05, **p < 0.01, and ***p < 0.001. ND = not detectable. Statistical significance was also calculated between each mutant using Welch’s t-test (File S1). The numbers on the top of the bar charts represent % wild type levels of activity or protein level for each sample

**Fig. 3:**
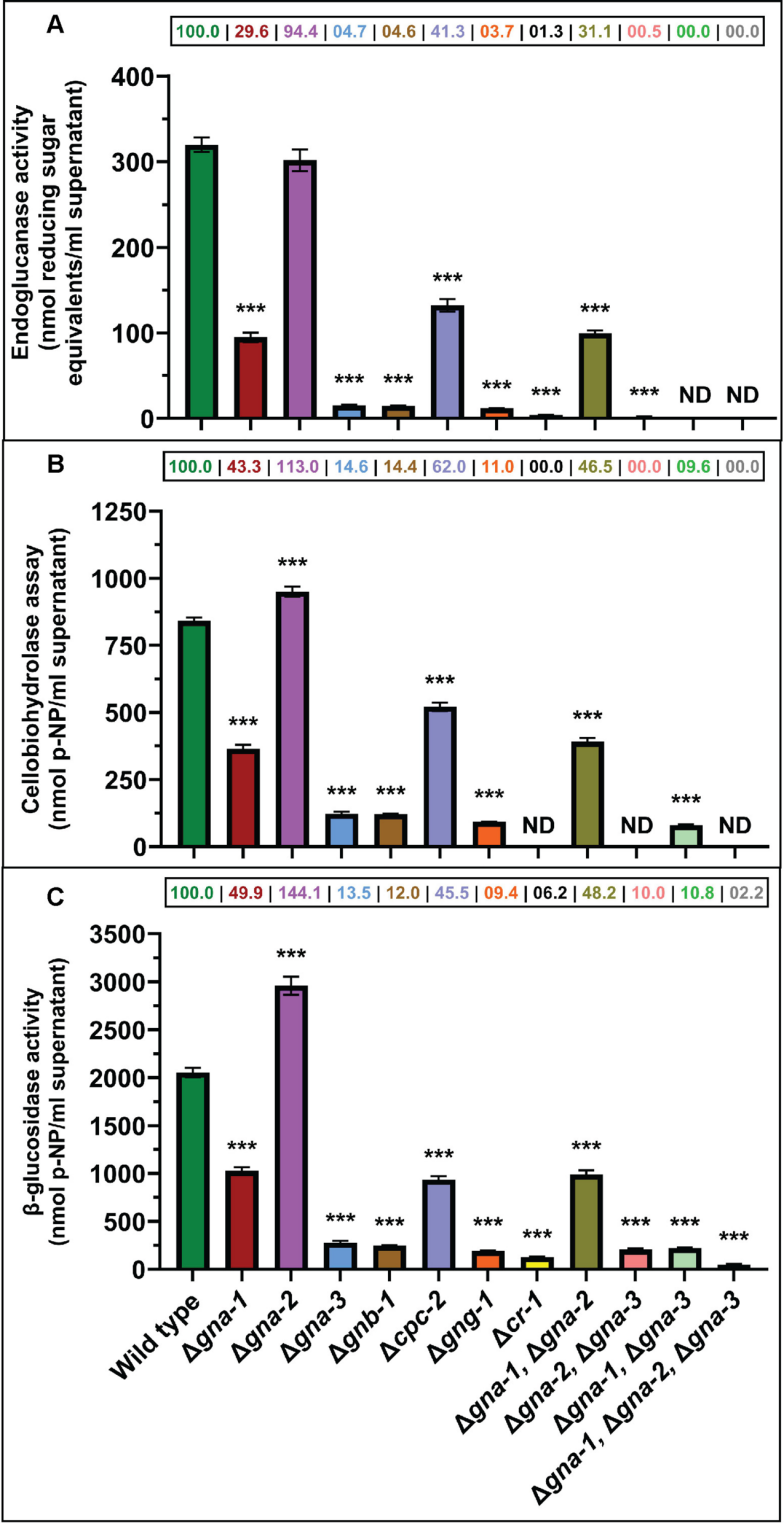
Activities of specific cellulase classes in G-protein single and multiple mutants, and the adenylyl cyclase mutant. Results for endoglucanase (**A**), cellobiohydrolase (**B**) and β-glucosidase (**C**) activity are shown. Strains were cultured and error analysis was performed as described in the legend for Fig. 2. Statistical significance was also calculated between each mutant using Welch’s t-test (File S1)

**Fig. 4:**
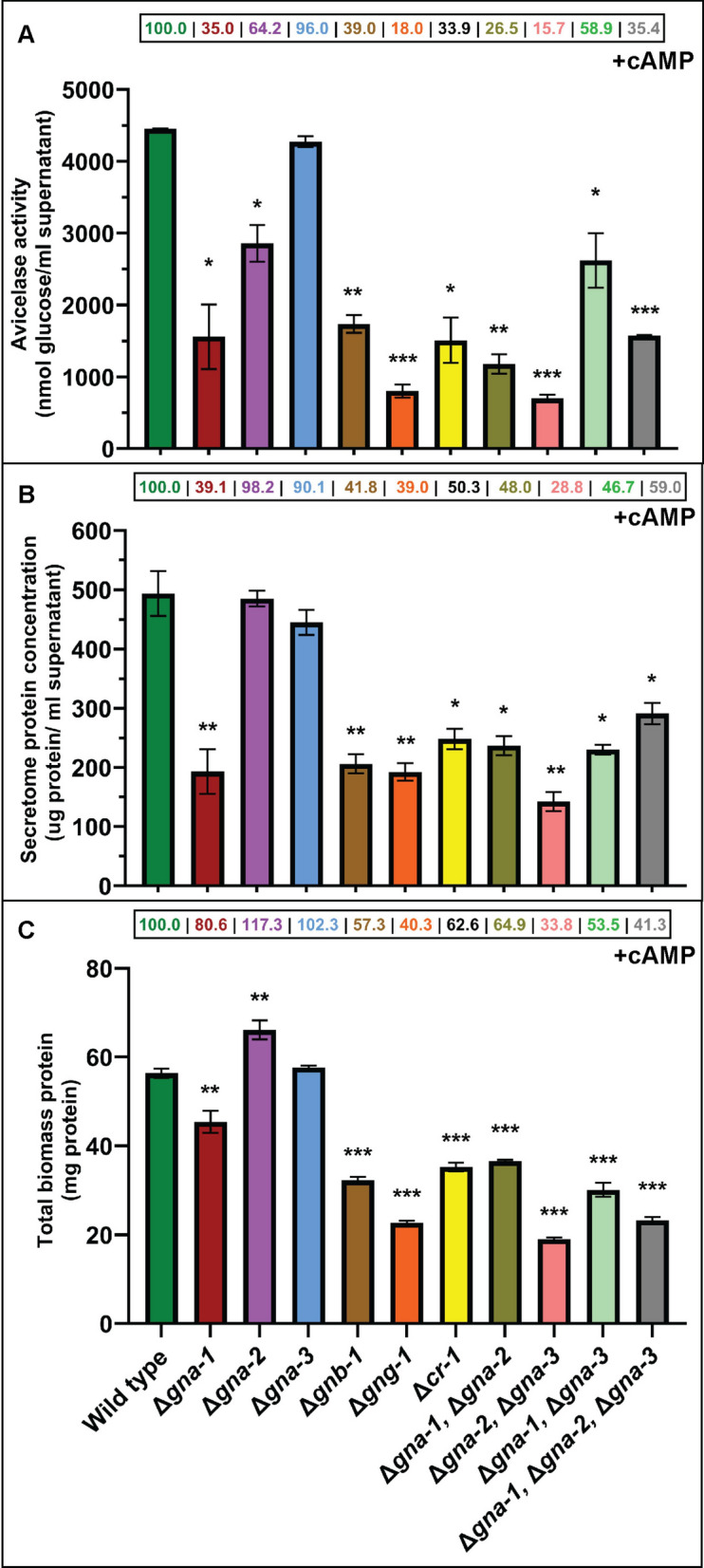
Effect of cAMP on Avicelase activity and protein levels in G-protein and adenylyl cyclase mutants. Avicelase activity and protein level measurements were performed as described in the legend to Fig. 2, except that all media were brought to a final concentration of 3 mM cAMP. **A** Represents Avicelase activity, **B** represents secretome protein concentration and **C** shows biomass protein levels. Error analysis was performed as described in the legend to Fig. 2. Statistical significance was also calculated between each mutant using Welch’s t-test (File S1)

**Fig. 5:**
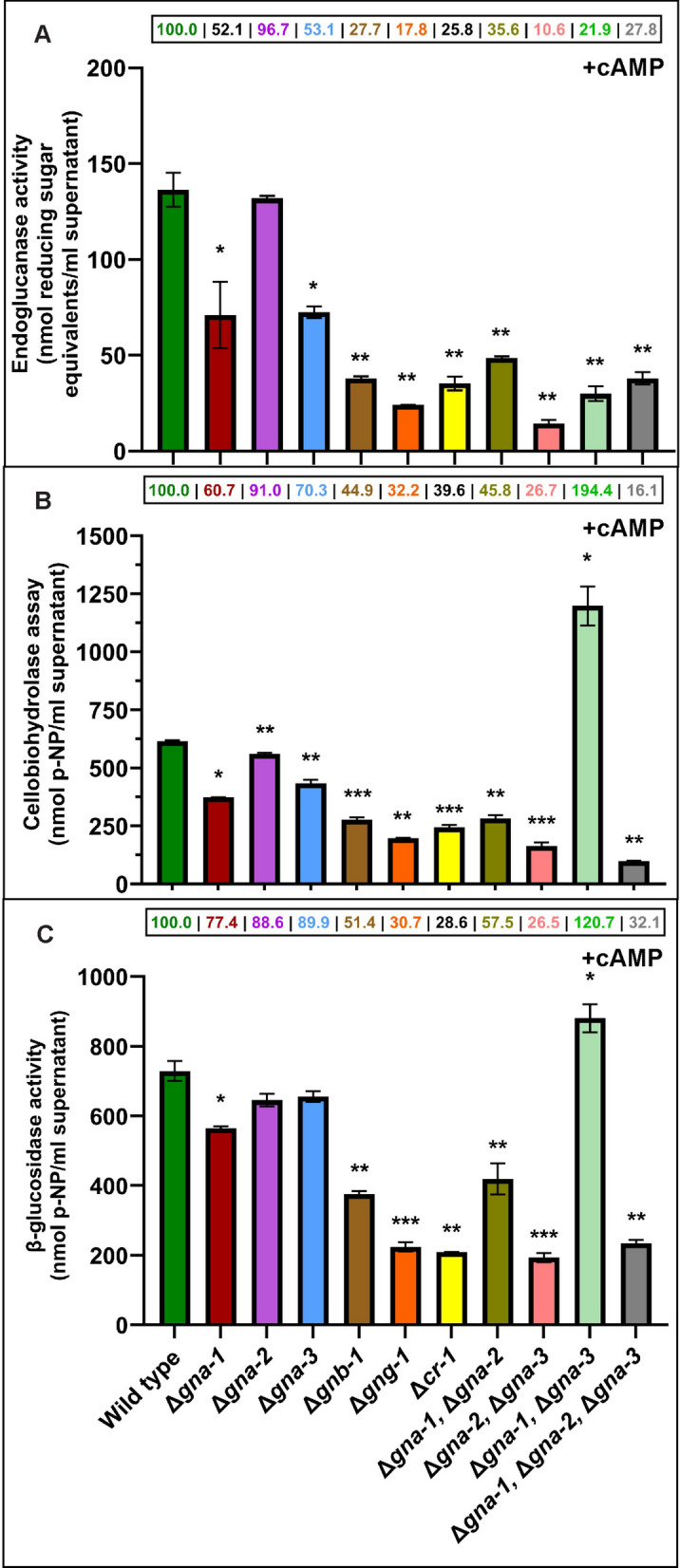
Effect of cAMP on activities of three enzyme classes in G-protein and adenylyl cyclase mutants. Results for endoglucanase (**A**), cellobiohydrolase (**B**) and β-glucosidase (**C**) activity are shown. Strains were cultured as described in the legend for Fig. 2, except that all media were brought to a final concentration of 3 mM cAMP. Error analysis was performed as described in the legend to Fig. 2. Statistical significance was also calculated between each mutant using Welch’s t-test (File S1)

For Figs. 2, 3, 4, 5, 7, and 8, the error bars reflect the Standard Error. The Q-test was performed on the technical replicate values to identify and remove any outliers based on the Z-values. For identifying significant differences in cellulase activities or protein levels, a pairwise, two-tailed Welch’s T-test was performed comparing activity numbers from each mutant to the wild type strain or to another mutant. p values for all comparisons are available in File S1.

## Results

### Expression of cellulase enzyme family genes is downregulated in Δ*gna-1*, Δ*gna-3*, and Δ*cr-1* mutants after transfer from glucose to cellulose

We recently published RNAseq analysis for wild type, Δ*gna-1*, Δ*gna-3*, and Δ*cr-1* strains after transfer from glucose to Avicel (cellulose) [9]. We identified 27 cellulase genes that were expressed to at least 10 transcripts per million (TPMs) in at least one strain on cellulose (Fig. 1). In the Δ*gna-1* mutant, expression of three endoglucanases was down-regulated by up to fivefold, the two major cellobiohydrolases were downregulated 2.8 and 4.3-fold, cellobiose dehydrogenase was downregulated 3.9-fold and seven polysaccharide monooxygenases were downregulated up to 5.4-fold. In contrast, five of the six expressed β-glucosidase family genes were not differentially expressed, and one intracellular β-glucosidase was up-regulated 1.8-fold (Fig. 1). Expression of cellulase genes showed a greater reduction in the Δ*gna-3* mutant, with four β-glucosidase genes and four endoglucanase genes downregulated up to 5.4-fold, all expressed cellobiose dehydrogenase genes reduced up to 4.7-fold, cellobiose dehydrogenase reduced 8.6-fold and nine polysaccharide monooxygenases downregulated up to 7.9-fold (Fig. 1). Expression of cellulase genes was most severely affected in the Δ*cr-1* mutant. All but one of the β-glucosidase genes was downregulated, expression of five endoglucanase genes was reduced up to 21.1-fold, three cellobiose dehydrogenase genes were downregulated up to 14.1-fold, cellobiose dehydrogenase expression was reduced by 18.9-fold, and levels of all expressed polysaccharide monooxygenases were reduced up to 41.1-fold (Fig. 1). These results demonstrate that the greatly reduced expression of multiple cellulase enzyme family genes is consistent with the relative Avicelase activity measured in the Δ*gna-1*, Δ*gna-3* and Δ*cr-1* strains [9].

Expanding on these results, we wanted to determine whether the transcriptional control of specific cellulase classes by G-protein subunits and adenylyl cyclase resulted in functional impairment of the activity of the resulting enzymes. A previous study from our group revealed combinatorial control of growth and development by multiple Gα subunits [19]. From this observation, we further hypothesized that multiple Gα subunits may act synergistically to regulate the activity of cellulase enzyme families in *N. crassa*. We thus included Gα double and triple mutants in this study [19]. We focused on Avicelase activity as an overall readout for cellulose degradation, and on the endoglucanase, cellobiohydrolase, and β-glucosidase enzyme families, as robust biochemical assays were available for these three specific enzyme classes [44].

### *gna-3*, *gnb-1*, *gng-1* and *cr-1* are essential for Avicelase activity

We grew wild type, the G-protein mutants, the Gα multiple mutants, and the adenylyl cyclase Δ*cr-1* mutant for 16 h in glucose, followed by two-days in Avicel and collected the cell-free supernatant (secretome) for analysis of Avicelase activity (see Methods; Fig. 2A). As observed previously [9, 10], the Δ*gna-2* mutant had wild type levels of Avicelase activity, activity was reduced to 63.1% of wild type in the Δ*gna-1* mutant, and activity was not detectable in the Δ*gna-3*, Δ*gnb-1*, Δ*gng-1* and Δ*cr-1* mutants (Fig. 2A). The Avicelase activity in the Δ*cpc-2* mutant was 82.3% of wild type (Fig. 2A). This contrasts with the reported lack of detectable activity in our earlier study [10], which likely resulted from mycelial clumping (fungal ball formation) [9, 21].

We also measured Avicelase activity in the mutants lacking multiple Gα subunit genes (Fig. 2A). Compared to wild type, the Δ*gna-1*, Δ*gna-2* (Δ1Δ2) double mutant had 45.7% of wild type Avicelase activity, which was also a significant reduction relative to Δ*gna-1* and Δ*gna-2* single mutants (File S1). The Δ*gna-2*, Δ*gna-3* (Δ2Δ3) and Δ*gna-1*, Δ*gna-3* (Δ1Δ3) double mutants, and the triple Gα mutant Δ1Δ2Δ3 displayed no detectable Avicelase activity, like the Δ*gna-3* single mutant (Fig. 2A). These results support our previous findings indicating that GNA-3 is essential for detectable Avicelase activity in *N. crassa* [10].

The secretome protein level in the Δ*gna-2* mutant and wild type strain was similar, while the amount in the Δ*cpc-2* mutant was 61.6% of wild type (Fig. 2B). The Δ1Δ2 double mutant had lower protein levels compared to the Δ*gna-1* single mutant (Fig. 2B; File S1). The Δ*gna-3*, Δ*gnb-1* and Δ*gng-1* mutants had greatly reduced secreted protein levels at ~ 12.7–18.4% of wild type, whereas the Δ*cr-1* mutants had only 9.2% (Fig. 2B). The Δ2Δ3 and the Δ1Δ3 double mutants possessed similar secreted protein levels at 13.8 and 15% of wild type, respectively (Fig. 2B; File S1). The Δ1Δ2Δ3 strain had the lowest protein secretion of all strains, at 5.9% of wild type.

The total biomass protein levels after growth on Avicel generally followed a similar trend to secretome protein levels for the strains (Fig. 2C; File S1). We also compared the biomass protein levels of the single knockout mutants during growth on glucose for 16 h to those observed after transfer to Avicel for two days (Fig. S1). The results showed that the biomass protein level trends in glucose cultures generally follow those for Avicel cultures. Two notable exceptions are the Δ*gnb-1* and Δ*gng-1* mutants (lacking components of the βγ dimer), which exhibit a greater decrease in biomass protein in Avicel vs. glucose relative to wild type than the other mutants.

### *gna-3 *works with *gna-1 *and *gna-2* during regulation of endoglucanase activity

The mutants followed a similar trend for endoglucanase activity as observed for Avicelase. The Δ*gna-2* mutant displayed wild type levels of endoglucanase activity, Δ*gna-1* and Δ*cpc-2* mutants had significantly reduced activity (29.6% and 41.3% of wild type, respectively), and the Δ*gna-3*, Δ*gnb-1*, and Δ*gng-1* mutants each exhibited ~ 4% of the endoglucanase activity of wild type (Fig. 3A). The adenylyl cyclase mutant Δ*cr-1* displayed barely detectable endoglucanase activity (1.3% of wild type) (Fig. 3A). These results and our RNAseq data demonstrate extensive regulation of the endoglucanase enzyme class by G-proteins and adenylyl cyclase (Fig. 1) [9]. The Δ1Δ2 double mutant had a similar level of endoglucanase activity as Δ*gna-1* (Fig. 3A, File S1). The Δ2Δ3 double mutant had endoglucanase activity only 0.5% of wild type, whereas the Δ1Δ3 double mutant and the triple mutant had no detectable activity (Fig. 3A), supporting *gna-1*, *gna-2* and *gna-3* as major co-regulators of endoglucanase activity.

### *gna-2 *and *gna-3* co-regulate cellobiohydrolase activity, while adenylyl cyclase is essential

The Δ*gna-3*, Δ*gnb-1*, and Δ*gng-1* mutants showed 11–14.6% wild type levels of cellobiohydrolase activity, while the Δ*gna-1* and Δ*cpc-2* mutants had 43.3% and 62% of wild type levels of activity, respectively (Fig. 3B). At 113% of the wild type, the Δ*gna-2* mutant possessed significantly elevated cellobiohydrolase activity (Fig. 3B). Cellobiohydrolase activity in the Δ1Δ2 double mutant was comparable to the Δ*gna-1* single mutant (46.5% of wild type; File S1). On the other hand, we observed that the Δ1Δ3 strain displayed lower levels of cellobiohydrolase activity than the Δ*gna-3* mutant (Fig. 3B, File S1). The Δ2Δ3 double mutant and Gα triple mutant had no detectable cellobiohydrolase activity. The extensive reduction in cellobiohydrolase gene expression in the Δ*cr-1* mutant and the lack of detectable cellobiohydrolase activity is consistent with the previous transcriptional analysis (Fig. 1) [9]. These results suggest that *gna-3* and *gna-2* are together required for cellobiohydrolase activity in *N. crassa*, and that CR-1 may act downstream of these two Gα subunits.

### Five of the six G-protein subunits and adenylyl cyclase are positive regulators of β-glucosidase activity

Similar to the results for cellobiohydrolase, the Δ*gna-2* mutant possesses elevated β-glucosidase activity (144.1% of wild type) (Fig. 3C). The Δ*gna-1* and Δ*cpc-2* mutants each have ~ 50% β-glucosidase activity as compared to wild type. It was interesting to observe that the β-glucosidase activity in the Δ*gna-1* mutant is 49.9% of wild type, since expression of extracellular β-glucosidase genes was not reduced in the Δ*gna-1* mutant at 4 h after the transfer to Avicel (Fig. 1). This suggested possible post-transcriptional regulation in the Δ*gna-1* mutant (see below). The Δ*gna-3*, Δ*gnb-1*, Δ*gng-1*, and Δ*cr-1* mutants have 9.4–13.5% β-glucosidase activity as compared to the wild type strain (Fig. 3C). None of the Gα multiple mutants showed a complete absence of detectable β-glucosidase activity (Fig. 3C). As for cellobiohydrolase activity, the Δ1Δ2 double mutant had ~ Δ*gna-1* levels of β-glucosidase activity (File S1). The Δ2Δ3 and Δ1Δ3 double mutants displayed β-glucosidase activity at levels lower than the Δ*gna-3* mutant (Fig. 3C, File S1). The triple Gα mutant had significantly reduced, and barely detectable activity (2.2%) as compared to wild type (Fig. 3C).

### Supplementation with cAMP influences Avicelase activity in several G protein mutants

Previous studies from our group have revealed roles for GNA-1 in regulating CR-1 enzyme activity and GNA-3 in modulating CR-1 protein levels [17, 20]. Our previous experiments with cultures transferred to Avicel supplemented with cAMP for 3-days demonstrated that Avicelase activity was restored to wild type levels in the Δ*gna-3* mutant [10], consistent with cAMP signaling as a downstream target of heterotrimeric G-proteins. Based on these findings, we investigated the effect of cAMP supplementation on the Avicelase activity of G-protein mutants and adenylyl cyclase mutant during a two-day transfer experiment on cellulose.

We observed reduced Avicelase activity in the Δ*gna-1*, Δ*gna-2*, and the Δ1Δ2 double mutants after cAMP addition (Figs. 2A, 4A). Similar to our previous study [10], the Avicelase activity in the Δ*gna-3* strain was fully rescued, and the Δ*gnb-1*, Δ*gng-1*, and Δ*cr-1* mutants were partially rescued by cAMP (Figs. 2A, 4A). We also observed low yet detectable Avicelase activity in the Δ2Δ3, Δ1Δ3, and Δ1Δ2Δ3 mutants with cAMP supplementation (Fig. 4A). The activity in the Δ2Δ3 double mutant was significantly lower than either single mutant with cAMP supplementation, but the Δ1Δ3 double mutant had Avicelase activity lower than Δ*gna-3* mutant and higher than the Δ*gna-1* mutant (Fig. 4A, File S1).

We did not note a significant difference in secreted protein concentration after cAMP supplementation in the wild type, Δ*gna-1*, Δ*gna-2*, and Δ1Δ2 double mutant strains (Figs. 2B, 4B, File S1). For the Δ*gna-1* mutant, we observed significantly reduced protein secretion compared to wild type upon cAMP addition, similar to our observation without cAMP supplementation (Figs. 2B, 4B, File S1). All the other G-protein single and multiple mutants experienced a significant rescue in protein concentration with cAMP addition, but in no case were protein levels restored to those of wild type (Fig. 4B, File S1). The trends in biomass protein were like those for secretome protein concentration for the strains (Fig. 4C). Notable exceptions were that the Δ*gna-2* mutant had higher biomass than wild type and the Δ1Δ2Δ3 triple mutant had less biomass protein than the Δ1Δ3 strain (Fig. 4C, File S1).

### cAMP supplementation rescues specific cellulase activities in several mutants

For endoglucanase activity, the wild type, Δ*gna-2*, and the Δ1Δ2 double mutants experienced a ~ 50% reduction in activity with cAMP supplementation (Fig. 5A), as compared to the wild type without supplementation (Fig. 3A). The Δ*gna-1* mutant had comparable endoglucanase activity with and without cAMP addition, suggesting that regulation by *gna-1* is cAMP-independent (File S1). The Δ*gna-3*, Δ*gnb-1*, and Δ*gng-1* mutants have 2–4 times greater endoglucanase activity with cAMP supplementation as without (Figs. 3A, 5A). The Δ*cr-1*, Δ2Δ3, Δ1Δ3, and Δ1Δ2Δ3 mutants, which had 0–1% endoglucanase activity without cAMP supplementation, had levels 10.6–27.8% of wild type with cAMP (Fig. 5A). These results support regulation of endoglucanase activity in Δ*gna-3*, Δ*gnb-1*, Δ*gng-1*, and Δ*cr-1* strains in a CR-1/cAMP-dependent manner.

The cellobiohydrolase activity was reduced in the wild type, Δ*gna-2*, and the Δ1Δ2 double mutants with cAMP addition (Figs. 3B, 5B, File S1). The Δ*gna-1* mutant had comparable cellobiohydrolase activity in ± cAMP conditions, at ~ 45% of the unsupplemented wild type strain (Fig. 5B, File S1). The Δ*gna-3*, Δ*gnb-1* and Δ*gng-1* mutants showed a 2–threefold increase in cellobiohydrolase activity with cAMP (Figs. 3B, 5B, File S1). The Δ*cr-1*, Δ2Δ3, and Δ1Δ2Δ3 mutants had non-detectable cellobiohydrolase activity under normal conditions but exhibited a rescue in activity after cAMP addition to up to 10–40% of wild type levels (Figs. 3B, 5B). Interestingly, the Δ1Δ3 double mutant possessed 194.4% of wild type activity upon cAMP supplementation, resembling the trend observed in the Δ*gna-3* mutant but to a greater extent. This result was not expected given that endoglucanase activity in the Δ1Δ3 mutant was 20% of wild type and suggests that the Δ1Δ3 mutant is exquisitely sensitive to cAMP with regards to cellobiohydrolase activity.

The β-glucosidase activity in the wild type, Δ*gna-1*, Δ*gna-2*, and the Δ1Δ2 double mutant strains was 20–36% of wild type without supplementation, and significantly reduced by at least ~ 50% as compared to their non-supplemented activity (Figs. 3C, 5C). The Δ*gna-3*, Δ1Δ3 and Δ1Δ2Δ3 strains showed a 2–fivefold increase in β-glucosidase activity upon cAMP supplementation (Figs. 3C, 5C), with the levels in the Δ1Δ3 mutant much higher than in wild type, like what was observed for cellobiohydrolase activity. The β-glucosidase activity in the Δ*gng-1* and Δ2Δ3 strains was not significantly affected by cAMP addition, suggesting that the β-glucosidase activity in these strains is regulated in a cAMP-independent manner (Figs. 3C, 5C, File S1).

### Expression of β-glucosidase genes cannot explain the reduced enzyme activity observed in the Δ*gna-1* mutant

**Fig. 6:**
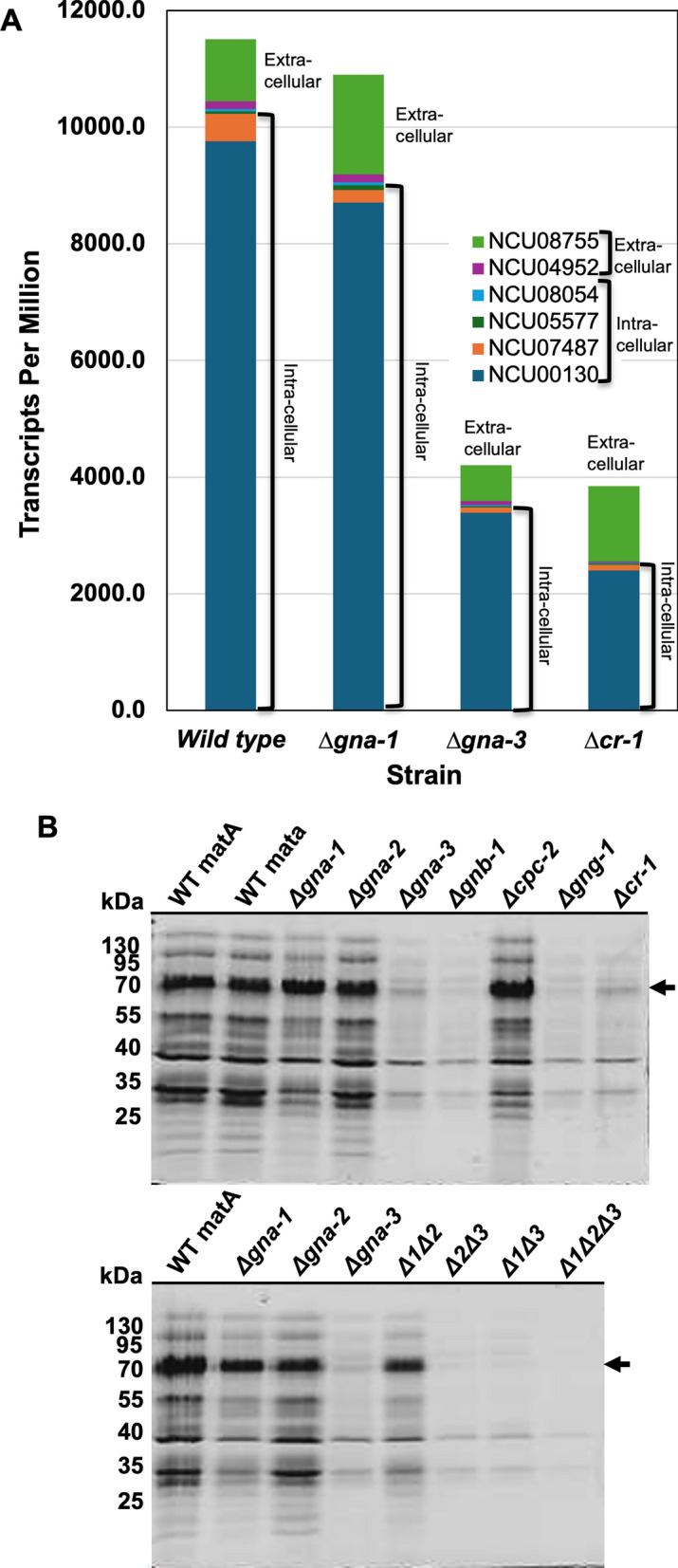
Expression of individual β-glucosidase genes and SDS-PAGE analysis of secretome samples. **A** Levels of β-glucosidase mRNAs in wild type, Δ*gna-1*, Δ*gna-3* and Δ*cr-1* strains. The average Transcripts Per Million (TPMs) for each of the six expressed β-glucosidase genes (out of seven total) were obtained from [9]. Only two β-glucosidase proteins are predicted to be secreted: NCU08755 and NCU04952. **B** SDS-PAGE of secreted proteins from strains lacking G protein subunit and adenylyl cyclase genes. A solution containing five-fold concentrated secretome protein samples (20 µl each) were electrophoresed using SDS-PAGE with a 10% resolving gel. The gel was Coomassie stained and positions of molecular weight markers determined (indicated on the left side of the gel). The predicted unmodified sizes of extracellular secreted β-glucosidase enzymes NCU08755 and NCU04952 are 95.6 kD and 77.7 kD, respectively. The position of the prominent band at ~ 70 kD is shown by the arrows

There are seven genes encoding β-glucosidase enzymes in *N. crassa*, with five predicted to be intracellular (NCU08054, NCU05577, NCU07487, NCU00130 and NCU03641) and two predicted as secreted proteins (NCU08755 and NCU04952) [42] (Fig. 6A). We observed that all genes except for NCU03641 are expressed during growth on cellulose [9] and NCU08755 mRNA is in large excess relative to NCU04952 in our strains (Fig. 6A). This is consistent with reports from other groups showing that NCU08755 is the major contributor to extracellular β-glucosidase activity in *N. crassa* [42].

Only β-glucosidase protein from NCU08755 and NCU04952 should be present in our samples, as we analyzed extracellular secreted protein. As noted above, β-glucosidase activity is reduced ~ 50% in the Δ*gna-1* mutant (Fig. 3C), even though expression of β-glucosidase genes is comparable to or even greater than observed in wild type (Figs. 1, 6A). This suggests some type of post-transcriptional regulation. To determine whether levels of extracellular β-glucosidase proteins were reduced in the Δ*gna-1* mutant, we subjected equal volumes of concentrated secretome samples to SDS-PAGE (Fig. 6B). We performed this analysis on all strains analyzed for β-glucosidase activity in Fig. 3C. As noted previously [10], the intensity of bands in the ~ 70 kDa size range generally follows the trend in Avicelase activity for the various strains (Fig. 6B). In this same earlier study, we showed that the major peptides in this region in wild type are the NCU07340/CBH-1 cellobiohydrolase and the NCU04952 β-glucosidase enzymes [10].

The predicted molecular masses of NCU08755 and NCU04952 are 95.6 and 77.7 kDa, respectively. Inspection of these regions after SDS-PAGE provided evidence for reduced intensity of the ~ 95.6 kDa, but not the 77.7 kDa β-glucosidase protein in the Δ*gna-1* mutant on both gels (Fig. 6B). These results are consistent with post-transcriptional control of β-glucosidase enzyme levels in the Δ*gna-1* strain.

### The activity of cellulase classes in RGS mutants and strains carrying constitutively active Gα alleles supports epistatic relationships

We previously measured Avicelase activity in the seven *N. crassa* RGS mutant strains after direct inoculation and two days growth in 2% Avicel cultures [6]. To directly compare results with those in this study, we grew cultures in 2% glucose and then transferred to 2% Avicel for two days (see Methods). We conducted assays on strains lacking each RGS gene, as well as strains carrying GTPase-deficient alleles of *gna-1* or *gna-3* (*gna-1*^Q204L^ and *gna-3*^Q208L^) expressed using a constitutive promoter. Since loss of a RGS protein or activation of its Gα target protein are both predicted to result in the same outcome, analysis of these strains would allow us to assign RGS proteins to GNA-1 or GNA-3.

**Fig. 7:**
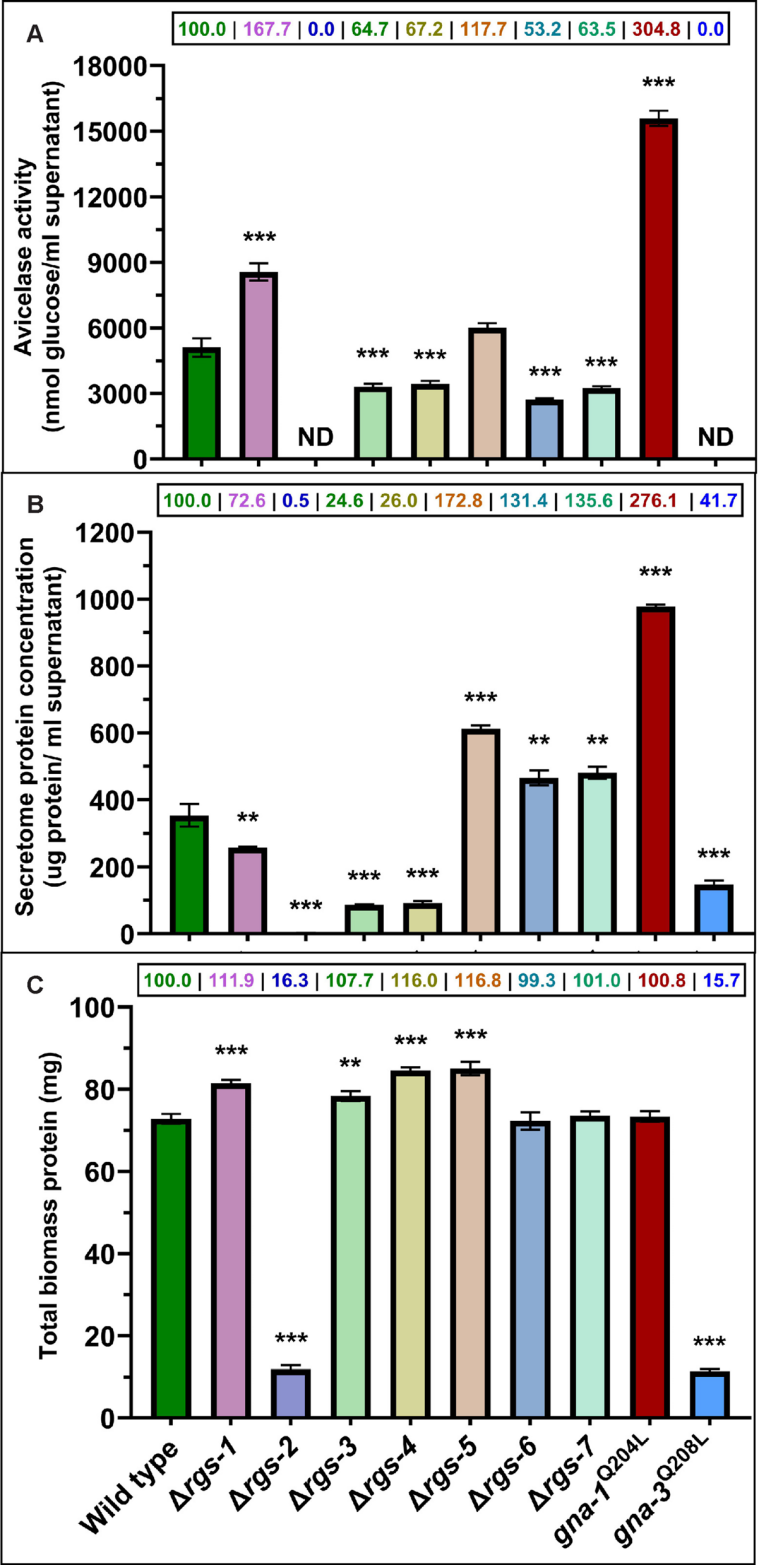
Avicelase activity and secretome and biomass protein levels in RGS mutants and strains expressing GTPase-deficient Gα gene alleles. Strains were cultured as described in the legend to Fig. 2. **A** Represents Avicelase activity, **B** represents secretome protein concentration and **C** shows biomass protein levels. Error analysis was performed as described in the legend to Fig. 2. Statistical significance was also calculated between each mutant using Welch’s t-test (File S1)

Consistent with our previous results, we observed that the Δ*rgs-1* strain had elevated Avicelase activity, that the Δ*rgs-3* strain had significantly lower activity, and the Δ*rgs-2* mutant had no detectable Avicelase activity, as compared to wild type (Fig. 7A). Using our new assay conditions, we also determined that Avicelase activity was not significantly different in the Δ*rgs-5* mutant, but reduced in the Δ*rgs-4,* Δ*rgs-6* and Δ*rgs-7* mutants, relative to the wild type strain (Fig. 7A). The strain expressing the *gna-1*^Q204L^ allele had significantly higher Avicelase activity (> 300% of wild type), whereas there was no detectable activity in the strain expressing the *gna-3*^Q208L^ allele (Fig. 7A). These findings support an epistatic relationship between *gna-1* and *rgs-1*, and between *gna-3* and *rgs-2* (and potentially *rgs-3*, *rgs-4*, *rgs-6* and *rgs-7*), for Avicelase activity.

We also measured the supernatant protein concentration and biomass protein in the nine strains (Fig. 7B, C). The secretome protein concentration in the Δ*rgs-1*, Δ*rgs-2*, Δ*rgs-3*, and Δ*rgs-4* mutants was significantly reduced, with levels in the Δ*rgs-2* mutant < 1% of wild type (Fig. 7B). On the contrary, the Δ*rgs-5*, Δ*rgs-6*, and Δ*rgs-7* mutants had significantly higher amounts of secreted protein than wild type (Fig. 7B). The *gna-1*^Q204L^ strain secreted ~ 2.8-fold more protein, whereas the *gna-3*^Q208L^ strain had levels of secreted protein only 41.7% of wild type (Fig. 7B). It was of interest that the secreted protein concentration did not always follow the same trend as Avicelase activity. For example, the higher Avicelase activity observed in the Δ*rgs-1* mutant was not accompanied by elevated levels of secreted protein. For biomass protein (Fig. 7C), levels were normal in the Δ*rgs-6*, Δ*rgs-7* and *gna-1*^Q204L^ strains. In contrast, elevated amounts relative to wild type were observed in Δ*rgs-1*, Δ*rgs-3*, Δ*rgs-4* and Δ*rgs-5* strains (Fig. 7C). As observed for other traits, biomass protein levels in the Δ*rgs-2* and *gna-3*^Q208L^ strains were greatly reduced relative to wild type (Fig. 7C).

**Fig. 8:**
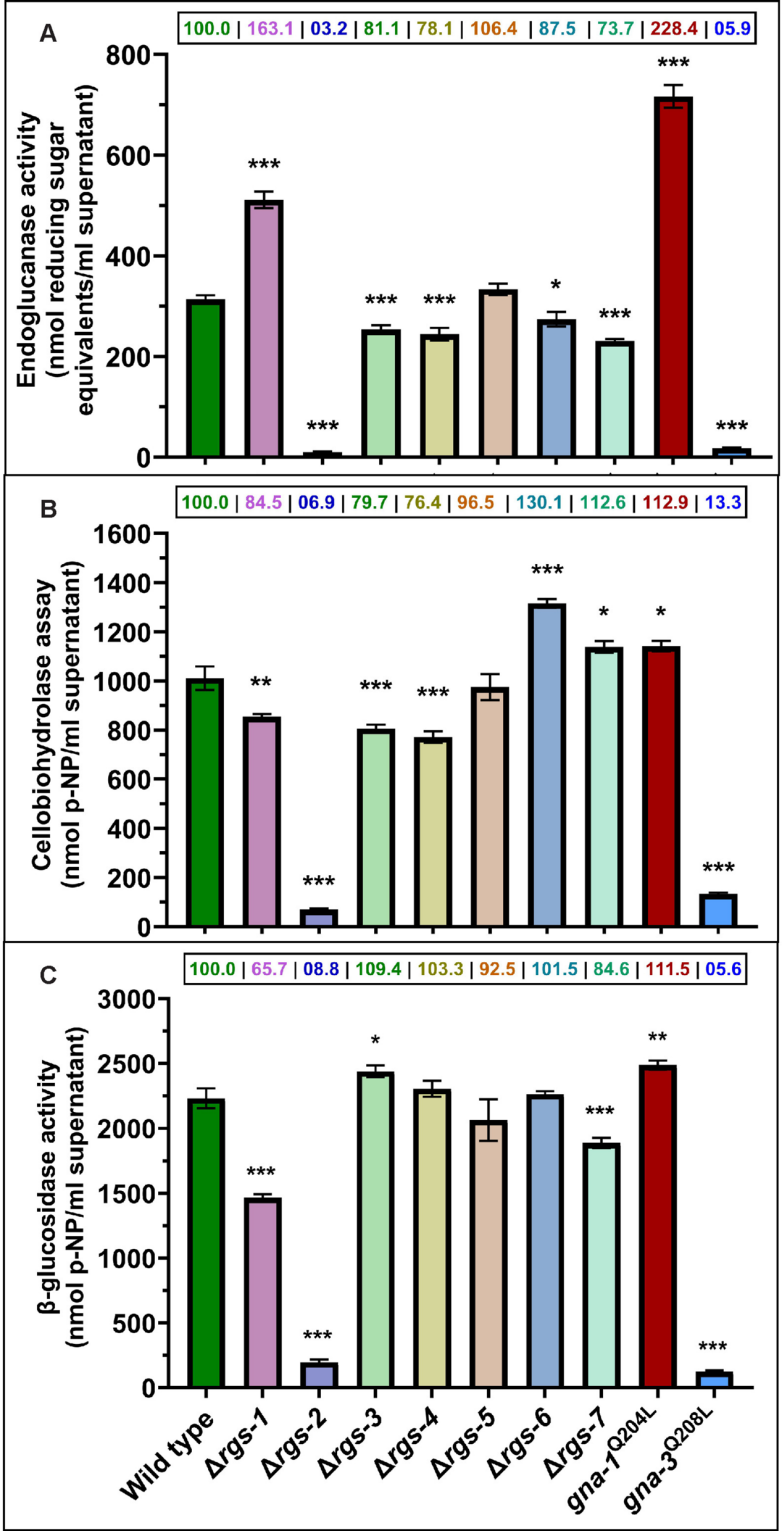
Activities of specific cellulase classes in RGS mutants and strains expressing constitutively active Gα alleles. Results for endoglucanase (**A**), cellobiohydrolase (**B**) and β-glucosidase (**C**) activity are shown. Strains were cultured and error analysis was performed as described in the legend for Fig. 2. Statistical significance was also calculated between each mutant using Welch’s t-test (File S1)

Following these results, we measured activities for the three specific enzyme classes in the RGS mutants and strains expressing the constitutively active Gα alleles. For the endoglucanase enzyme class, we found that the Δ*rgs-1* and *gna-1*^Q204L^ strains had significantly higher activity than wild type at 163.1% and 228.4%, respectively (Fig. 8A). This suggests an epistatic relationship between *gna-1* and *rgs-1* for endoglucanase activity in *N. crassa*. Among the other strains, Δ*rgs-2* had only 3.2% the activity of wild type, while Δ*rgs-3*, Δ*rgs-4*, Δ*rgs-6*, and Δ*rgs-7* had significantly reduced endoglucanase activity, at levels 73.7%−87.5% of wild type (Fig. 8A). The *gna-3*^Q208L^ strain displayed 5.9% wild type activity, thus strongly supporting an epistatic relationship between *gna-3* and *rgs-2* for endoglucanase activity (Fig. 8A).

Upon measuring the cellobiohydrolase activity of these strains, we observed that Δ*rgs-6* and Δ*rgs-7* mutants had ~ 130% and 113% activity of wild type, respectively, and that the *gna-1*^Q204L^ strain had 112.9% wild type activity (Fig. 8B). This may point to RGS-6 and RGS-7 acting on GNA-1 to regulate cellobiohydrolase activity. On the contrary, the Δ*rgs-2* and *gna-3*^Q208L^ strains had 6.9% and 13.3% of wild type cellobiohydrolase activity, respectively (Fig. 8B). This supports an epistatic relationship between *gna-3* and *rgs-2*, much like already observed for Avicelase and endoglucanase activities. The Δ*rgs-1*, Δ*rgs-3*, and Δ*rgs-4* mutants exhibited significantly reduced activity that was ~ 76–85% of wild type (Fig. 8B).

Levels of extracellular β-glucosidase activity were normal in the Δ*rgs-4*, Δ*rgs-5* and Δ*rgs-6* strains (Fig. 5C). The Δ*rgs-1*, Δ*rgs-2*, Δ*rgs-7* and *gna-3*^Q208L^ strains had significantly reduced β-glucosidase activity, with levels in the Δ*rgs-2* and *gna-3*^Q208L^ strains ~ 6–9% wild type activity (Fig. 8C). Thus, like what was observed for other enzymes, *gna-3* and *rgs-2* demonstrated an epistatic relationship for β-glucosidase activity. In contrast, levels of β-glucosidase activity were elevated relative to wild type in the Δ*rgs-3* and *gna-1*^Q204L^ strains (Fig. 8C), suggesting a genetic relationship between *gna-1* and *rgs-3* for this enzyme class.

## Discussion

**Fig. 9:**
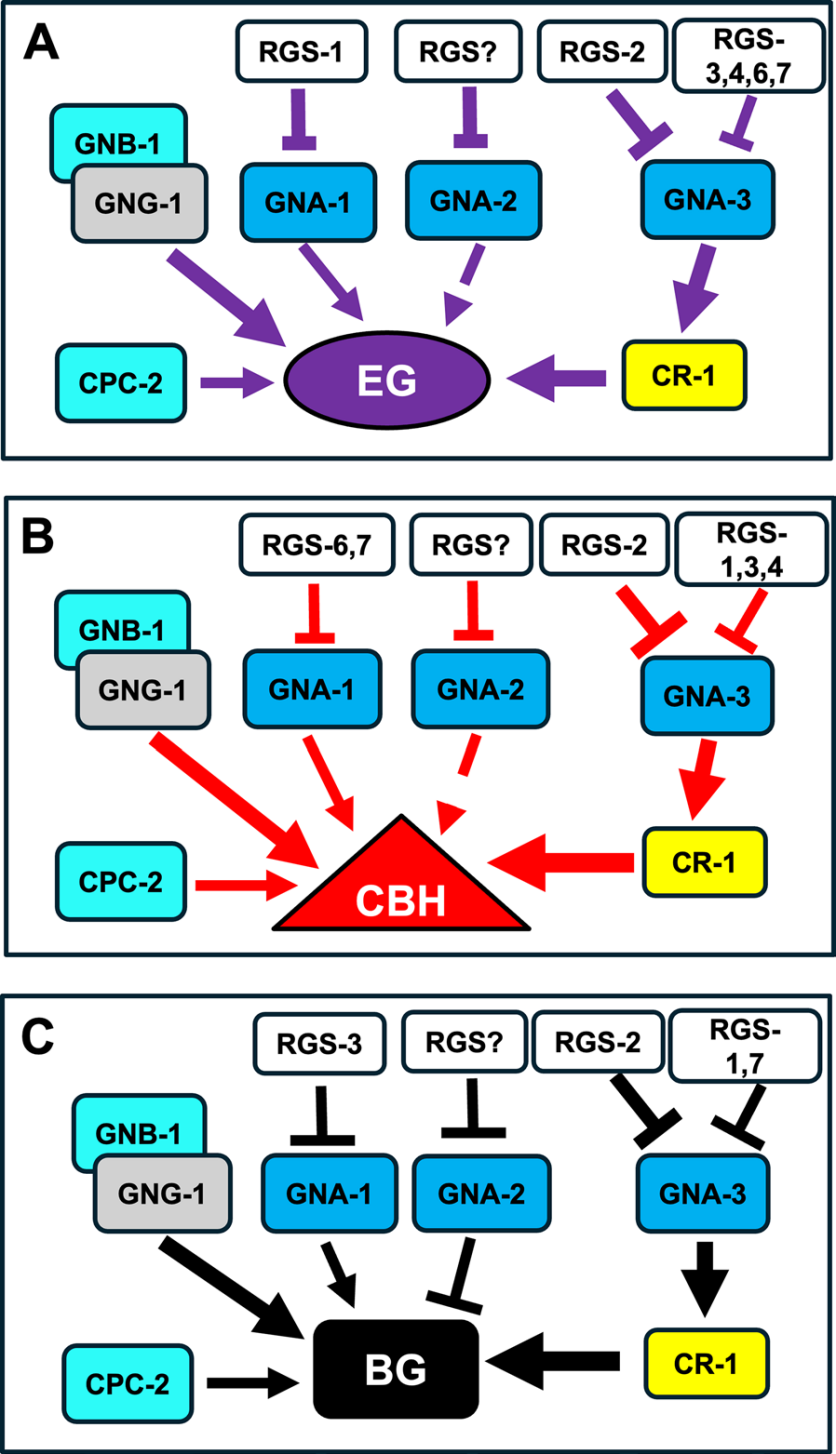
A model for regulation of three cellulase classes by G protein signaling components. The seven RGS proteins are represented in white, the three Gα proteins are shown in blue, the Gβ proteins are colored in turquoise (GNB-1 and CPC-2), the Gγ subunit is represented in gray (GNG-1), and the adenylyl cyclase (CR-1) is indicated in yellow. Impacts on subsequent proteins are illustrated by colored arrows. Thin solid arrows indicate loss of some activity in a single mutant and thick solid arrows represent severe or complete loss of activity in a single mutant, and broken arrows indicate that an effect is only observed when that gene is mutated in combination with another Gα gene. Supporting data can be found in Figs. 3 and 8. **A** Endoglucanase activity. Effects on endoglucanase activity (EG) are shown using purple arrows. EG activity is positively regulated by CR-1, GNB-1, GNG-1, CPC-2, and by the combined action of GNA-1 and GNA-3 or GNA-2 and GNA-3. RGS-1 and RGS-2 are strong negative regulators acting upstream of GNA-1 and GNA-3, respectively. RGS-3,4,6 and 7 are more minor negative regulators of GNA-3. The RGS acting upstream of GNA-2 is unknown. **B** Cellobiohydrolase activity. Impacts on cellobiohydrolase activity (CBH) are shown using red arrows. CR-1 alone, and GNA-2 and GNA-3 acting together, are essential for CBH activity. Although GNA-2 is a weak negative regulator of CBH activity on its own, loss of *gna-2* synergizes to reduce activity in a Δ*gna-1*, Δ*gna-3* double mutant. CBH activity is positively regulated by GNA-1, GNA-3, GNB-1, GNG-1 and CPC-2. RGS-6 and RGS-7 are negative regulators acting upstream of GNA-1. RGS-2 is a strong negative regulator, while RGS-1,3 and 4 are more minor negative regulators, of GNA-3. The RGS acting upstream of GNA-2 is unknown. **C** β-glucosidase activity. Effects on β-glucosidase activity (BG) are illustrated using black arrows. CR-1, GNA-1, GNA-3, GNB-1, GNG-1, and CPC-2 are positive regulators of BG activity. Although GNA-2 is a negative regulator of BG activity, its absence negatively synergizes with loss of GNA-1 in the double mutant. BG activity is greatly reduced in the absence of all three Gα proteins. RGS-2 is a strong negative regulator, while RGS-1 and 7 are more minor negative regulators, of GNA-3. RGS-3 functions as a negative regulator of GNA-1. The RGS proteins acting upstream of GNA-2 are unknown

Our overall goal is to engineer *N. crassa* to overproduce different classes of cellulase enzymes, through perturbation of environmental sensing pathways involving heterotrimeric G proteins. This current study was focused on determining the activity for three cellulase enzyme families in various G-protein signaling pathway mutants. Other groups have previously measured specific cellulase enzyme activities in the secretomes of fungal wild type and mutant strains (e.g., [8, 37, 44]). We add to this research area by conducting a comprehensive study to elucidate the complex regulation of cellulase activity in *N. crassa* mediated by heterotrimeric G-proteins, the effector protein adenylyl cyclase/CR-1, and the RGS proteins (see model in Fig. 9). Our work reveals that the activity of various cellulase enzyme families is indeed regulated through specific G-protein signaling pathway proteins. The Δ*gna-1*, Δ*gna-3* double mutant showed a complete loss of endoglucanase activity, thus supporting co-regulation by GNA-1 and GNA-3. The adenylyl cyclase *cr-1* was found to be essential for detectable cellobiohydrolase activity. This may result from co-regulation of CR-1 and cellobiohydrolase activity by the Gα subunits *gna-2* and *gna-3*, as the double mutant lacking these genes also showed no cellobiohydrolase activity. Supplementation with cAMP rescued Avicelase activity in all G protein mutants with nondetectable activity, indicating that cAMP signaling is a major pathway for cellulase regulation. Cellobiohydrolase and β-glucosidase enzyme activities were especially sensitive to cAMP in the Δ*gna-1*, Δ*gna-3* double mutant, suggesting an interesting regulatory mechanism in this strain. A caveat to this study is that we have not quantified the cellobiose dehydrogenase and polysaccharide monooxygenase activity and these activities would also contribute to the overall Avicelase activity for the strains.

Different RGS genes acted as positive and negative regulators of the activities of specific cellulase classes (Fig. 9). The RGS mutants Δ*rgs-3* and Δ*rgs-4* behaved similarly for all specific enzyme activities and had comparable secretome protein concentrations. The *gna-1*^Q204L^ strain displayed significantly higher activity for all specific enzyme classes, Avicelase activity, and secreted protein concentration. Interestingly, the high Avicelase activity of the Δ*rgs-1* strain seemed to majorly result from the increased endoglucanase activity, since the other cellulase activities were significantly reduced compared to wild type. On the other hand, the *gna-3*^Q208L^ strain and the Δ*rgs-2* mutant had nearly identical, significantly reduced levels for specific cellulase activities, and no detectable Avicelase activity. The only difference between these two strains was that the secretome protein concentration was significantly lower in the Δ*rgs-2* mutant, suggesting greater misregulation of protein secretion than observed with constitutive activation of the *gna-3* gene.

In a recent study [9], we showed how the expression of an important cellulase transcription factor CLR-2 is downregulated in two G-protein mutants. Based on the results for activities of specific enzyme classes, these enzyme families are regulated differently by the same G-proteins. It is intriguing that G-protein subunits regulate *clr-2* expression, which in turn regulates cellulases, and yet the control is not uniform across the various cellulase families. This suggests a role for post-translational regulation of cellulase proteins. We provide preliminary evidence for post-transcriptional regulation of extracellular β-glucosidases in the Δ*gna-1* mutant, as the normal expression of extracellular β-glucosidase genes in this strain does not correlate with the observed ~ 50% reduction in activity or the decreased intensity of the species in the predicted size range for one of the secreted β-glucosidase enzymes after SDS-PAGE. This could result from decreased translation, secretion/protein glycosylation or increased protein turnover, or a combination of these effects.

## Conclusions

Our current work demonstrates that G protein signaling components operate mostly as positive regulators of cellulase enzyme production/secretion in *N. crassa*. Analysis of strains carrying GTPase deficient alleles of *gna-1* and *gna-3* revealed that activated GNA-1 leads to higher levels of Avicelase, endoglucanase, cellobiohydrolase and β-glucosidase activity. In contrast, GNA-3^Q208L^ behaves similarly to the Δ*gna-3* null allele, with low activity for all assays; we previously provided evidence that this behavior was due to tethering of GNA-3 by the Gβ subunit GNB-1 [10]. Considering these points, GNA-1^Q204L^ could be used for strain engineering to overproduce cellulases. Likewise, since loss of *rgs-1* lead to elevated endoglucanase activity, this mutation could be “stacked” with *gna-1*^Q204L^ to further increase production of this enzyme class. Finally, overexpression or mutational activation of adenylyl cyclase and other components of cAMP signaling are additional prospective targets for increased cellulase production in *N. crassa*.

## Supplementary Information

Supplementary material 1.

Supplementary material 2.

Supplementary material 3.

## Data Availability

All data supporting the findings of this study are available within the paper.

## References

[1] Alqinyah M, Hooks SB. Regulating the regulators: epigenetic, transcriptional, and post-translational regulation of RGS proteins. Cell Signal. 2018;42:77–87. 10.1016/j.cellsig.2017.10.007. 29042285 10.1016/j.cellsig.2017.10.007 PMC5732043

[2] Barr BK, Hsieh YL, Ganem B, Wilson DB. Identification of two functionally different classes of exocellulases. Biochemistry. 1996;35(2):586–92. 10.1021/bi9520388. 8555231 10.1021/bi9520388

[3] Beeson WT, Vu VV, Span EA, Phillips CM, Marletta MA. Cellulose degradation by polysaccharide monooxygenases. Annu Rev Biochem. 2015;84:923–46. 10.1146/annurev-biochem-060614-034439. 25784051 10.1146/annurev-biochem-060614-034439

[4] Bischof RH, Ramoni J, Seiboth B. Cellulases and beyond: the first 70 years of the enzyme producer *Trichoderma reesei*. Microb Cell Fact. 2016;15(1):106. 10.1186/s12934-016-0507-6. 27287427 10.1186/s12934-016-0507-6 PMC4902900

[5] Bosnjak N, Smith KM, Asaria I, Lahola-Chomiak A, Kishore N, Todd AT, et al. Involvement of a G protein regulatory circuit in alternative oxidase production in *Neurospora crassa*. G3 Genes|Genomes|Genetics. 2019;9(10):3453–65. 10.1534/g3.119.400522. 31444295 10.1534/g3.119.400522 PMC6778808

[6] Cabrera IE, Oza Y, Carrillo AJ, Collier LA, Wright SJ, Li L, et al. Regulator of G protein signaling proteins control growth, development and cellulase production in *Neurospora crassa*. J Fungi. 2022. 10.3390/jof8101076. 10.3390/jof8101076 PMC9604755 36294641

[7] Cantarel BL, Coutinho PM, Rancurel C, Bernard T, Lombard V, Henrissat B. The Carbohydrate-Active EnZymes database (CAZy): an expert resource for glycogenomics. Nucleic Acids Res. 2009;37(Database issue):D233–8. 10.1093/nar/gkn663. 18838391 10.1093/nar/gkn663 PMC2686590

[8] Christopher M, Sreeja-Raju A, Abraham A, Gokhale DV, Pandey A, Sukumaran RK. Early cellular events and potential regulators of cellulase induction in *Penicillium janthinellum* NCIM 1366. Sci Rep. 2023;13(1):5057. 10.1038/s41598-023-32340-x. 36977777 10.1038/s41598-023-32340-x PMC10050438

[9] Collier L, Oza Y, Quinn M, Carrillo AJ, Campbell MM, Borkovich KA. Heterotrimeric G-proteins and cAMP regulate gene expression during growth on cellulose in *Neurospora crassa*. MBio. 2026. 10.1128/mbio.03720-25. 41537596 10.1128/mbio.03720-25 PMC12892961

[10] Collier LA, Ghosh A, Borkovich KA. Heterotrimeric G-Protein signaling is required for cellulose degradation in *Neurospora crassa*. MBio. 2020;11(6):e02419-02420. 33234686 10.1128/mBio.02419-20 PMC7701987

[11] Coradetti ST, Craig JP, Xiong Y, Shock T, Tian C, Glass NL. Conserved and essential transcription factors for cellulase gene expression in ascomycete fungi. Proc Natl Acad Sci U S A. 2012;109(19):7397–402. 10.1073/pnas.1200785109. 22532664 10.1073/pnas.1200785109 PMC3358856

[12] Coradetti ST, Xiong Y, Glass NL. Analysis of a conserved cellulase transcriptional regulator reveals inducer-independent production of cellulolytic enzymes in *Neurospora crassa*. Microbiol Open. 2013;2(4):595–609. 10.1002/mbo3.94. 10.1002/mbo3.94 PMC3948607 23766336

[13] Fu C, Iyer P, Herkal A, Abdullah J, Stout A, Free SJ. Identification and characterization of genes required for cell-to-cell fusion in *Neurospora crassa*. Eukaryot Cell. 2011;10(8):1100–9. 10.1128/EC.05003-11. 21666072 10.1128/EC.05003-11 PMC3165452

[14] Garud A, Carrillo AJ, Collier LA, Ghosh A, Kim JD, Lopez-Lopez B, et al. Genetic relationships between the RACK1 homolog *cpc-2* and heterotrimeric G protein subunit genes in *Neurospora crassa*. PLoS ONE. 2019;14(10):e0223334. 10.1371/journal.pone.0223334. 31581262 10.1371/journal.pone.0223334 PMC6776386

[15] Glass NL, Schmoll M, Cate JH, Coradetti S. Plant cell wall deconstruction by ascomycete fungi. Annu Rev Microbiol. 2013;67:477–98. 10.1146/annurev-micro-092611-150044. 23808333 10.1146/annurev-micro-092611-150044

[16] Huberman LB, Coradetti ST, Glass NL. Network of nutrient-sensing pathways and a conserved kinase cascade integrate osmolarity and carbon sensing in *Neurospora crassa*. Proc Natl Acad Sci U S A. 2017;114(41):E8665–74. 10.1073/pnas.1707713114. 28973881 10.1073/pnas.1707713114 PMC5642704

[17] Ivey FD, Yang Q, Borkovich KA. Positive regulation of adenylyl cyclase activity by a Galphai homolog in *Neurospora crassa*. Fungal Genet Biol. 1999;26(1):48–61. 10.1006/fgbi.1998.1101. 10072319 10.1006/fgbi.1998.1101

[18] Jung MG, Kim SS, Yu JH, Shin KS. Characterization of gprK encoding a putative hybrid G-protein-coupled receptor in *Aspergillus fumigatus*. PLoS ONE. 2016;11(9):e0161312. 10.1371/journal.pone.0161312. 27584150 10.1371/journal.pone.0161312 PMC5008803

[19] Kays AM, Borkovich KA. Severe impairment of growth and differentiation in a *Neurospora crassa* mutant lacking all heterotrimeric G alpha proteins. Genetics. 2004;166(3):1229–40. 15082543 10.1534/genetics.166.3.1229 PMC1470763

[20] Kays AM, Rowley PS, Baasiri RA, Borkovich KA. Regulation of conidiation and adenylyl cyclase levels by the Galpha protein GNA-3 in *Neurospora crassa*. Mol Cell Biol. 2000;20(20):7693–705. 11003665 10.1128/mcb.20.20.7693-7705.2000 PMC86343

[21] Kelliher CM, Loros JJ, Dunlap JC. Evaluating the circadian rhythm and response to glucose addition in dispersed growth cultures of *Neurospora crassa*. Fungal Biol. 2020;124(5):398–406. 10.1016/j.funbio.2019.11.004. 32389302 10.1016/j.funbio.2019.11.004 PMC7217977

[22] Kolde R (2025) pheatmap: Pretty Heatmaps.

[23] Krystofova S, Borkovich KA. The heterotrimeric G-protein subunits GNG-1 and GNB-1 form a Gbetagamma dimer required for normal female fertility, asexual development, and galpha protein levels in *Neurospora crassa*. Eukaryot Cell. 2005;4(2):365–78. 10.1128/EC.4.2.365-378.2005. 15701799 10.1128/EC.4.2.365-378.2005 PMC549333

[24] Li L, Borkovich KA. GPR-4 is a predicted G-protein-coupled receptor required for carbon source-dependent asexual growth and development in *Neurospora crassa*. Eukaryot Cell. 2006;5(8):1287–300. 10.1128/EC.00109-06. 16896213 10.1128/EC.00109-06 PMC1539153

[25] Liu Q, Li J, Gao R, Li J, Ma G, Tian C. CLR-4, a novel conserved transcription factor for cellulase gene expression in ascomycete fungi. Mol Microbiol. 2019;111(2):373–94. 10.1111/mmi.14160. 30474279 10.1111/mmi.14160

[26] Liu S, Anderson PJ, Rajagopal S, Lefkowitz RJ, Rockman HA. G protein-coupled receptors: a century of research and discovery. Circ Res. 2024;135(1):174–97. 10.1161/CIRCRESAHA.124.323067. 38900852 10.1161/CIRCRESAHA.124.323067 PMC11192237

[27] Mattam AJ, Chaudhari YB, Velankar HR. Factors regulating cellulolytic gene expression in filamentous fungi: an overview. Microb Cell Fact. 2022;21(1):44. 10.1186/s12934-022-01764-x. 35317826 10.1186/s12934-022-01764-x PMC8939176

[28] Phillips CM, Beeson WT, Cate JH, Marletta MA. Cellobiose dehydrogenase and a copper-dependent polysaccharide monooxygenase potentiate cellulose degradation by *Neurospora crassa*. ACS Chem Biol. 2011;6(16):1399–406. 10.1021/cb200351y. 22004347 10.1021/cb200351y

[29] Ross EM, Wilkie TM. GTPase-activating proteins for heterotrimeric G proteins: regulators of G protein signaling (RGS) and RGS-like proteins. Annu Rev Biochem. 2000;69:795–827. 10.1146/annurev.biochem.69.1.795. 10966476 10.1146/annurev.biochem.69.1.795

[30] Ruel K, Nishiyama Y, Joseleau JP. Crystalline and amorphous cellulose in the secondary walls of *Arabidopsis*. Plant Sci. 2012;193–194:48–61. 10.1016/j.plantsci.2012.05.008. 22794918 10.1016/j.plantsci.2012.05.008

[31] Saharay M, Guo H, Smith JC. Catalytic mechanism of cellulose degradation by a cellobiohydrolase, CelS. PLoS ONE. 2010;5(10):e12947. 10.1371/journal.pone.0012947. 20967294 10.1371/journal.pone.0012947 PMC2953488

[32] Schmoll M. Light, stress, sex and carbon - the photoreceptor ENVOY as a central checkpoint in the physiology of *Trichoderma reesei*. Fungal Biol. 2018;122(6):479–86. 10.1016/j.funbio.2017.10.007. 29801792 10.1016/j.funbio.2017.10.007

[33] Schmoll M, Schuster A, Silva RN, Kubicek CP. The G-alpha protein GNA3 of *Hypocrea jecorina* (anamorph *Trichoderma reesei*) regulates cellulase gene expression in the presence of light. Eukaryot Cell. 2009;8(3):410–20. 10.1128/EC.00256-08. 19136572 10.1128/EC.00256-08 PMC2653238

[34] Seibel C, Gremel G, do Nascimento Silva R, Schuster A, Kubicek CP, Schmoll M. Light-dependent roles of the G-protein α subunit GNA1 of *Hypocrea jecorina* (anamorph *Trichoderma reesei*). BMC Biol. 2009;7(1):58. 10.1186/1741-7007-7-58. 19728862 10.1186/1741-7007-7-58 PMC2749820

[35] Sygmund C, Kracher D, Scheiblbrandner S, Zahma K, Felice AK, Harreither W, et al. Characterization of the two *Neurospora crassa* cellobiose dehydrogenases and their connection to oxidative cellulose degradation. Appl Environ Microbiol. 2012;78(17):6161–71. 10.1128/AEM.01503-12. 22729546 10.1128/AEM.01503-12 PMC3416632

[36] Team RC. R: A language and environment for statistical computing. Vienna, Austria: R Foundation for Statistical Computing; 2021.

[37] Tian C, Beeson WT, Iavarone AT, Sun J, Marletta MA, Cate JH, et al. Systems analysis of plant cell wall degradation by the model filamentous fungus *Neurospora crassa*. Proc Natl Acad Sci U S A. 2009;106(52):22157–62. 10.1073/pnas.0906810106. 20018766 10.1073/pnas.0906810106 PMC2794032

[38] Turner GE, Borkovich KA. Identification of a G protein alpha subunit from *Neurospora crassa* that is a member of the Gi family. J Biol Chem. 1993;268(20):14805–11. 8325859

[39] van den Brink J, de Vries RP. Fungal enzyme sets for plant polysaccharide degradation. Appl Microbiol Biotechnol. 2011;91(6):1477–92. 10.1007/s00253-011-3473-2. 21785931 10.1007/s00253-011-3473-2 PMC3160556

[40] Vogel HJ. Distribution of lysine pathways among fungi: evolutionary implications. Am Nat. 1964;98:435–46.

[41] Wu VW, Thieme N, Huberman LB, Dietschmann A, Kowbel DJ, Lee J, et al. The regulatory and transcriptional landscape associated with carbon utilization in a filamentous fungus. Proc Natl Acad Sci U S A. 2020;117(11):6003–13. 10.1073/pnas.1915611117. 32111691 10.1073/pnas.1915611117 PMC7084071

[42] Wu W, Kasuga T, Xiong X, Ma D, Fan Z. Location and contribution of individual beta-glucosidase from *Neurospora crassa* to total beta-glucosidase activity. Arch Microbiol. 2013;195(12):823–9. 10.1007/s00203-013-0931-5. 24162785 10.1007/s00203-013-0931-5

[43] Xiong Y, Sun J, Glass NL. VIB1, a link between glucose signaling and carbon catabolite repression, is essential for plant cell wall degradation in *Neurospora crassa*. PLoS Genet. 2014;10(8):e1004500. 25144221 10.1371/journal.pgen.1004500 PMC4140635

[44] Xue F, Zhao Z, Gu S, Chen M, Xu J, Luo X, et al. The transcriptional factor Clr-5 is involved in cellulose degradation through regulation of amino acid metabolism in *Neurospora crassa*. BMC Biotechnol. 2023;23(1):50. 10.1186/s12896-023-00823-4. 38031036 10.1186/s12896-023-00823-4 PMC10687990

[45] Yang Q, Poole SI, Borkovich KA. A G-protein beta subunit required for sexual and vegetative development and maintenance of normal G alpha protein levels in *Neurospora crassa*. Eukaryot Cell. 2002;1(3):378–90. 12455986 10.1128/EC.1.3.378-390.2002 PMC118013

[46] Yenkie KM, Wu W, Clark RL, Pfleger BF, Root TW, Maravelias CT. A roadmap for the synthesis of separation networks for the recovery of bio-based chemicals: matching biological and process feasibility. Biotechnol Adv. 2016;34(8):1362–83. 10.1016/j.biotechadv.2016.10.003. 27756578 10.1016/j.biotechadv.2016.10.003

[47] Zhang H, Tang W, Liu K, Huang Q, Zhang X, Yan X, et al. Eight RGS and RGS-like proteins orchestrate growth, differentiation, and pathogenicity of *Magnaporthe oryzae*. PLoS Pathog. 2011;7(12):e1002450. 10.1371/journal.ppat.1002450. 22241981 10.1371/journal.ppat.1002450 PMC3248559

[48] Znameroski EA, Coradetti ST, Roche CM, Tsai JC, Iavarone AT, Cate JH, et al. Induction of lignocellulose-degrading enzymes in *Neurospora crassa* by cellodextrins. Proc Natl Acad Sci U S A. 2012;109(16):6012–7. 10.1073/pnas.1118440109. 22474347 10.1073/pnas.1118440109 PMC3341005

