# Supplementary material for: G-protein signaling components differentially regulate the activity of multiple cellulase enzyme classes in Neurospora crassa
Source: Biotechnol Biofuels Bioprod. 2026 Jun 5;19:61. doi: 10.1186/s13068-026-02779-x (PMC13459781; doi:10.1186/s13068-026-02779-x)
Supplement: Supplementary file 1 [file 13068_2026_2779_MOESM3_ESM.docx]

**Table S1. Strains used in this study.**

| **Strain** | **Relevant Genotype** | **Name as Referenced in Text** | **Relevant Gene Number** | **Source/**  **Reference #** |
| --- | --- | --- | --- | --- |
| 74-OR23-1A | Wild type, *mat A* | Wild type |  | FGSC^1^ |
| 3b10 | *∆gna*-*1*::*hph, mat a* | *∆gna*-*1* | NCU06493 | (1) |
| 12378 | ∆*gna*-*2*::*hph, mat A* | ∆*gna*-*2* | NCU06729 | FGSC |
| 31c2 | ∆*gna*-*3*::*hph, mat A* | ∆*gna*-*3* | NCU05206 | (2) |
| 42-8-3 | ∆*gnb*-*1*::*hph, mat A* | ∆*gnb*-*1* | NCU00440 | (3) |
| 5-5-3 | ∆*gng*-*1*::*hph, mat A* | ∆*gng*-*1* | NCU00041 | (4) |
| cpc-2-5 | ∆*cpc*-*2*::*hph, mat A* | ∆*cpc*-*2* | NCU05810 | (5) |
| ∆cr-1 | ∆*cr*-*1*::*hph, mat a* | ∆*cr*-*1* | NCU08377 | (6) |
| rgs-1-2a | ∆*rgs-1::hph, mat a* | ∆*rgs-1* | NCU08319 | (7) |
| rgs-2-7a | ∆*rgs-2::hph, mat a* | ∆*rgs-2* | NCU05435 | (7) |
| rgs-3-2a | ∆*rgs-3::hph, mat a* | ∆*rgs-3* | NCU08343 | (7) |
| rgs-4-7A | ∆*rgs-4::hph, mat A* | ∆*rgs-4* | NCU03937 | (7) |
| rgs-5-3 | ∆*rgs-5::hph, mat a* | ∆*rgs-5* | NCU09883 | (7) |
| rgs-6-a | ∆*rgs-6::hph, mat a* | ∆*rgs-6* | NCU03153 | (7) |
| rgs-7-A | ∆*rgs-7::hph, mat A* | ∆*rgs-7* | NCU09415 | (7) |
| B3 | ∆*gna-1::hph*, ∆*gna-2::pyrG*^+^, *mat a* | ∆1∆2 |  | (8) |
| g1.3 | ∆*gna-1::hph*, ∆*gna-3::hph*, *mat A* | ∆1∆3 |  | (8) |
| 2.3g | *gna-2::pyrG*^+^, ∆*gna-3::hph*, *mat A* | ∆2∆3 |  | (8) |
| noa | ∆*gna-1::hph*, *gna-2::pyrG*^+^, ∆*gna-3::hph*, *mat A* | ∆1∆2∆3 |  | (8) |
| ∆1gna-1* | ∆*gna*-*1*::*hph, gna*-*1^Q204L^*::*his*-*3^+^, mat A* | *gna*-*1^Q204L^* |  | (6) |
| gna3Q208L | p*ccg*-*1*::*gna*-*3^Q208L^*::*his*-*3^+^, mat A* | *gna*-*3^Q208L^* |  | (6) |

1. FGSC = Fungal Genetics Stock Center (9)

**TABLE S1 REFERENCES**

1.	**Ivey FD, Yang Q, Borkovich KA.** 1999. Positive regulation of adenylyl cyclase activity by a Galphai homolog in *Neurospora crassa*. Fungal Genet Biol **26:**48-61.

2.	**Kays AM, Rowley PS, Baasiri RA, Borkovich KA.** 2000. Regulation of conidiation and adenylyl cyclase levels by the Galpha protein GNA-3 in *Neurospora crassa*. Mol Cell Biol **20:**7693-7705.

3.	**Yang Q, Poole SI, Borkovich KA.** 2002. A G-protein beta subunit required for sexual and vegetative development and maintenance of normal G alpha protein levels in *Neurospora crassa*. Eukaryot Cell **1:**378-390.

4.	**Krystofova S, Borkovich KA.** 2005. The heterotrimeric G-protein subunits GNG-1 and GNB-1 form a Gbetagamma dimer required for normal female fertility, asexual development, and galpha protein levels in *Neurospora crassa*. Eukaryot Cell **4:**365-378.

5.	**Garud A, Carrillo AJ, Collier LA, Ghosh A, Kim JD, Lopez-Lopez B, Ouyang S, Borkovich KA.** 2019. Genetic relationships between the RACK1 homolog *cpc-2* and heterotrimeric G protein subunit genes in *Neurospora crassa*. PLoS One **14:**e0223334.

6.	**Collier LA, Ghosh A, Borkovich KA.** 2020. Heterotrimeric G-Protein Signaling Is Required for Cellulose Degradation in *Neurospora crassa*. mBio **11:**e02419-02420.

7.	**Cabrera IE, Oza Y, Carrillo AJ, Collier LA, Wright SJ, Li L, Borkovich KA.** 2022. Regulator of G Protein Signaling Proteins Control Growth, Development and Cellulase Production in Neurospora crassa. J Fungi (Basel) **8**.

8.	**Kays AM, Borkovich KA.** 2004. Severe impairment of growth and differentiation in a *Neurospora crassa* mutant lacking all heterotrimeric G alpha proteins. Genetics **166:**1229-1240.

9.	**McCluskey K, Wiest A, Plamann M.** 2010. The Fungal Genetics Stock Center: a repository for 50 years of fungal genetics research. J Biosci **35:**119-126.
